# Central Nervous System Tuberculosis in Immunocompromised Patients: A Case Report Emphasizing Immune Status and Early Recognition and Treatment

**DOI:** 10.7759/cureus.52715

**Published:** 2024-01-22

**Authors:** Marta Quaresma, Madalena Paulino, Ana Oliveira, Ana Nunes

**Affiliations:** 1 Internal Medicine Department, Hospital Vila Franca Xira, Vila Franca Xira, PRT; 2 Internal Medicine Department, Hospital de São José, Centro Hospitalar Universitário de Lisboa Central, Lisboa, PRT

**Keywords:** hiv, imunocompromised, cns tuberculosis, tuberculosis, cns infections

## Abstract

Tuberculosis (TB) remains a global health challenge. Although pulmonary TB is the most frequent presentation, extrapulmonary involvement can occur, especially in immunocompromised patients. HIV-positive individuals are particularly vulnerable to opportunistic infections, such as TB, and CNS involvement is more prevalent in these patients, often leading to a poorer prognosis. CNS TB management is challenging due to nonspecific symptoms and delayed diagnosis, contributing to high mortality. It can manifest diffusely as tuberculous meningitis (TBM), localized as tuberculoma or tuberculous abscess, or as extradural and intradural spinal infections. TBM is the primary CNS manifestation, bearing significant morbidity and mortality, and rarely complicates with involvement of the spinal cord, termed tuberculous myelitis, which is associated with an unfavorable prognosis.

A 61-year-old male, smoker with a history of substance abuse, undergoing seven months of antiretroviral therapy (ART) for HIV-1, presented with a two-day history of altered consciousness, sphincter incontinence, and fever. He also reported headaches, dizziness, and sleep disturbances over the past months. The examination revealed fever, asthenia, prostration, disorientation, neck rigidity, and bilateral lower limb weakness. Initial tests indicated lymphopenia, hyponatremia, and a slightly elevated C-reactive protein. Cranial CT showed no abnormalities. Lumbar puncture yielded abnormal cerebrospinal fluid (CSF), xanthochromic, hyperproteinorrheic (2316 g/L), hypoglycorrhagic (24mg/dl), with pleocytosis predominantly of mononuclear cells (98%). Compared to the values prior to ART treatment, the patient had a decreased HIV-1 viral (44 copies/ml) load but also a decreased CD4+ cell count (43 cells/mm^3^). Given the patient's rapid clinical deterioration, immunosuppression history, and a positive interferon-gamma release assay (IGRA) prior to ART, treatment with antituberculous drugs and dexamethasone was started at admission. Subsequently, *Mycobacterium tuberculosis* was identified through polymerase chain reaction (PCR) of the CSF. Cranial and spinal MRI revealed leptomeningeal enhancement from C2-C3 to the cauda equina, consistent with meningitis, without intracranial extension, and findings suggestive of myelitis, without evidence of tuberculomas or spinal cord osseous involvement. One week after treatment, the recovery of higher neurological functions became evident. Improvement in lower limb motor deficits had a delayed trajectory, with marginal progress observed at discharge. After an eight-week incubation, CSF mycobacterial culture analysis yielded negative results.

This case discusses the importance of early suspicion and intervention in CNS infection prognosis. Attention to signs and symptoms beyond the most frequent ones is crucial, particularly in immunocompromised individuals like HIV patients. Identifying CSF features in different CNS infections and group-specific particulars facilitates the prompt initiation of treatment. Additionally, in co-infected patients (HIV and CNS TB), considering factors such as ART duration, CD4+ cell count, and viral load is important, in influencing the disease's incidence, course, and prognosis.

## Introduction

HIV-induced immunosuppression markedly elevates susceptibility to opportunistic infections, including tuberculosis (TB) [1]. Additionally, HIV-infected patients are more likely to have extrapulmonary TB (EPTB) including lymph nodes, bones, and the central nervous system (CNS) [2]. HIV co-infected patients are five times more likely to develop CNS TB, associated with poor clinical outcomes [3]. Factors contributing to increased EPTB prevalence in HIV patients include immune system compromise, reduced CD4+ cell count, heightened risk of TB dissemination, delayed EPTB diagnosis, and severe complications [4]. This risk is increased with more advanced levels of immunosuppression since cell-mediated immunity is critical to control hematogenous spread through macrophages and, consequently, the disease disseminates readily in HIV-infected individuals with reduced CD4+ cell count [5].

Mitigating opportunistic infections such as TB/EPTB in HIV-infected patients involves the early management of latent TB infection, strengthening the immune system via antiretroviral therapy (ART), and vigilant monitoring for TB symptoms [6]. This group of patients carries a 10% annual risk of progression to active infection, with increasing risk as the CD4+ cell count declines, against the 10-20% lifetime risk of developing TB in non-infected individuals [4]. In those already under ART, most studies report higher rates of death at six to nine months after initiation of antituberculosis treatment in HIV-infected patients (24-67%) compared to HIV-uninfected patients (0-30%) [7]. An interaction between low CD4+ cell count, high viral load, and increased risk and severity of TB has been described [8,9]. Effective ART, TB preventive therapy, early diagnosis of TB, and prompt management, effectively mitigate this risk and improve outcomes [9,10].

CNS TB occurs in 1-5% of all patients with TB and 10% of those with HIV-related TB [11]. It can be manifested diffusely as tuberculous meningitis (TBM) (70-80% of cases), localized as tuberculoma or tuberculous abscess, or in extradural and intradural spinal infections [12]. Known manifestations include headache, fever, neck stiffness, and altered mental status in TBM; neurological deficits, seizures, and changes in mental status in tuberculomas; and neurological deficits like muscle weakness, numbness, pain, sensory disturbances, and bladder or bowel control issues, in tuberculous myelitis [4,12].

Manifestations of CNS TB are subtle and less specific in HIV-infected patients especially with low CD4+ cell count, culminating in delayed diagnosis, treatment, and potentially worse outcomes [1]. An altered level of consciousness may be more prominent in TBM in those HIV-infected individuals [4]. A comprehensive approach including TB history, radiological and cerebrospinal fluid (CSF) analysis, and, in some cases, biopsy is necessary in these cases [9]. Early diagnosis and prompt management, effectively improve outcomes and reduce the severity of neurologic complications in CNS TB [10]. In these cases, anti-TB treatment may extend beyond one year due to the potential for a slow therapeutic response [10].

## Case presentation

A 61-year-old male was admitted to our hospital due to a state of altered consciousness, sphincter incontinence, and fever. The patient had been evaluated in the previous day for prostration, and blood tests had revealed hyponatremia (serum sodium concentration of 123 mmol/L) and a slight elevation (1.11mg/dl) in C-reactive protein (CRP). He was discharged with discontinuation of lorazepam, considering hyponatremia a side effect, and started empirical antibiotherapy with ciprofloxacin 500 mg every 12 hours to address an infectious focus needing further investigation. Furthermore, he had a history of a respiratory infection three months prior, which had been treated with two courses of antibiotics, followed by persistent symptoms such as headache, dizziness, and sleep disturbances.

The patient was a 45-pack-year smoker, had a previous history of intravenous drug use, and had recently been diagnosed with HIV-1 infection, for which he had been on antiretroviral therapy (ART) with Biktarvy® (tenofovir alafenamide, emtricitabine, bictegravir, 50/200/25 mg) for seven months. His latest follow-up results showed a CD4+ cell count of 114 cells/mm^3^, a viral load of 146.053 copies/ml, and a positive interferon-gamma release assay (IGRA) test. The patient also had a history of depression and was receiving lorazepam in addition to ART.

Upon physical examination, the patient presented with a fever (38°C), reduced alertness, disorientation, and incoherent speech. He was hemodynamically stable, with no signs of respiratory distress or need for oxygen. Neurologically, he exhibited reduced vigilance, disorientation in terms of time, place, and person with fluent but confused speech, and psychomotor slowing. His upper limb muscle tone and strength were maintained, but there was reduced strength in the lower limbs (2/5 on the Oxford muscle grading scale). Nuchal rigidity was observed. The remaining physical examination was unremarkable. Analytical results at admission revealed lymphopenia (830 cells/μL), hyponatremia (mmol/L), and a CRP of 1.57 mg/dL (Table 1).

**Table 1:** Analytical results BUN: blood urea nitrogen; EBV: Epstein-Barr virus; HIV: human immunodeficiency virus 1; HLA B57: human leukocyte antigen B57; PCR: polymerase chain reaction; VCA: viral capsid antigen

Blood analysis	Results	Reference value
Haemoglobin (g/dl)	13	13 -17
Haematocrit (%)	27.9	40 – 50
White blood cell count (10^3^/µl)	4.6	4 -10
Neutrophils (10^3^/µl)	3.1	2 - 7
Lymphocytes (10^3^/µl)	0.8	1.26-3.35
Platelets (10^3^/µl)	363	150 - 400
Folic Acid (ng/mL)	2.5	>5.38
Ferritin (ng/mL)	344	22-322
Serum Creatinine (mg/dl)	0.75	0.70 – 1.30
BUN (mg/dl)	58.8	< 50
Sodium (mmol/L)	127	136 -145
Potassium (mmol/L)	4.75	3.5 -5.1
Chloride (mmol/L)	88	98 -107
Reactive C Protein (mg/dl)	1.57	0.06 -10
Sedimentation Velocity	101	0-15
Aspartate Aminotransferase (UI/L)	25	15-37
Alanine Aminotransferase (UI/L)	17	16-63
Gamma-glutamyl Transferase (UI/L)	34	15-85
Alkaline Phosphatase (UI/L)	55	50-136
Total Bilirubin (mg/dl)	0.46	<1
Creatine Kinase (CK) (mg/dl)	63	39 - 308
PCR SARS-COV2	Negative	
Hepatitis A, B, and C	Negative	
VIH1 antibodies	Positive	
HLA B57	Negative	
CD4+ cell count (cells/mm^3^)	43	40- 230
CD4/CD8 ratio	0.31	3.30- 19.40
HIV Viral Load (copies /ml)	44	5.71- 26.30
Anti-Epstein Barr antibodies	Negative	
IgM anti-EBV anti-VCA antibodies	Negative	
IgG anti-EBV anti-VCA antibodies	Positive	
Anti-EBV nuclear antigen (EBNA-G) IgG	Negative	

A brain CT scan was performed, which ruled out acute ischemic or hemorrhagic vascular injury and other structural abnormalities. A lumbar puncture was performed, and CSF with a xantchromic and turbid appearance was obtained (Table 2). CSF analysis showed pleocytosis with a white blood cell count of 203 cells/uL, primarily consisting of mononuclear cells (98%) and adenosine deaminase (ADA) of 62.04 U/L. Elevated protein levels (2316 mg/dL) and decreased glucose levels (24 mg/dL) in the CSF, with a CSF:serum glucose ratio of 0.2, supported the diagnosis of bacterial meningitis (Table 2).

**Table 2:** Cerebrospinal Fluid analysis at admission and 72 hours after admission ADA: adenosine deaminase; BK: Koch's bacillus; CSF: cerebrospinal fluid; HSV: herpes simplex virus; JC virus: John Cunningham virus; PCR: polymerase chain reaction; VDRL:  Venereal Disease Research Laboratory; VZV: varicella zoster virus

Cerebrospinal fluid analysis	Admission	72 hours after admission	Reference value
Appearance	Xantochromic	Turbid; Xantochromic	
Protein (mg/dL)	2316	3191	15 - 45
Glucose (mg/dL)	24	29	> 40
CSF: serum glucose ratio	0.23	0,24	
White Cell Count (Cel/uL)	203	1038	< 5
Differential	Predominately mononuclear cells (98%)	Predominately mononuclear cells (99%)	
VDRL	Negative		
ADA (U/L)	62.04		< 9
BK PCR	Positive		
JC virus PCR	Negative		
IgM HSV-1	Negative		
IgG HSV-1	Positive		
IgM VZV	Negative		
*Cryptococcus neoformans* Antigen	Negative		
CSF culture	Negative		

Due to the rapid neurological deterioration, the patient's history of immunosuppression, a positive IGRA before the initiation of ART, and certain CSF characteristics, such as hyperproteinorrachia with a concentration of 2316 mg/dL and an ADA level of 62.04 U/L, the possibility of tuberculous meningoencephalitis had to be considered. The direct examination with acid-fast bacilli (AFB) search in the CSF was not performed.

Treatment with antitubercular medication (HRZE (isoniazid, rifampin, pyrazinamide, and ethambutol) and pyridoxine were initiated, along with dexamethasone (0.4 mg/kg/day). Additionally, treatment with acyclovir was maintained for nine days until viral etiology was definitively ruled out through CSF analysis. ART was continued, excluding bictegravir and tenofovir alafenamide. Instead, a regimen comprising Kivexa® (abacavir + lamivudine, 600 mg/300 mg) along with dolutegravir 100 mg was initiated. Subsequent CSF results revealed the presence of BK virus via polymerase chain reaction (PCR) while excluding other viral pathogens (Table 2). A second lumbar puncture performed after 72 hours revealed CSF with similar macroscopic characteristics but an increased white blood cell count (1038 cells/uL), maintaining predominance of mononuclear cells, further increased protein levels (3191 mg/dL), and hypoglycorrhachia (29 mg/dL) (Table2).

**Figure 1:**
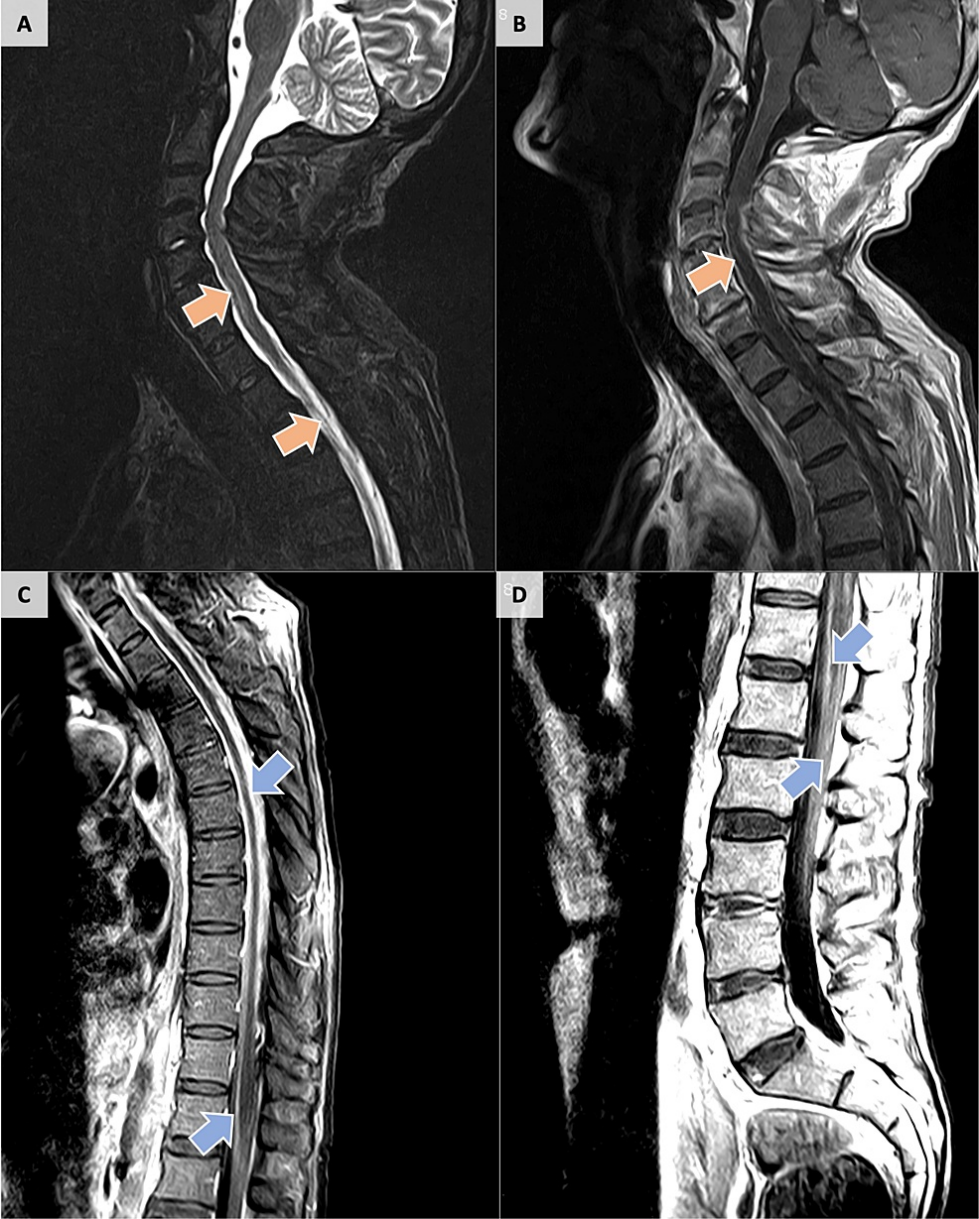
Magnetic Resonance Imaging of spinal cord (A) Sagittal T2 STIR and (B) Sagittal T1 GAD: high T2 signal in the anterior aspect of cervical and thoracic spinal cord, with multifocal T1 gadolinium enhancement. (C) Sagittal T1 GAD fatsat thoracic spine and (D) Sagittal T1 GAD lumbar spine: pachymeningeal and leptomeningeal T1 gadolinium enhancement from C2/3 to cauda equina. STIR: short tau inversion recovery; GAD: gadolinium contrast; fatsat: fat-saturation

Following an eight-week incubation period, the mycobacterial culture analysis of the CSF revealed a negative result (Table 2). Further analysis of the immunologic state of the patient demonstrated a CD4+ cell count of 43 cells/mm^3^ and a viral load of 44 copies/mL (Table 1). Chest CT showed no lung lesions or invitations. Cranioencephalic and neuroaxis MRI revealed patchy and leptomeningeal enhancement on T1-weighted images from C2-C3 to the cauda equina, consistent with meningitis, without intracranial extension, possibly TBM, considering the clinical data. Additionally, there was anterior intramedullary T2 hypersignal in the cervicodorsal region with several foci of enhancement, suggestive of tuberculous myelitis, without tuberculomas or bone marrow involvement (Figure 1).

After one week of treatment, the patient started showing a progressive improvement in upper neurological functions, and a slight improvement in lower limb weakness. The tapering of corticosteroid therapy began two weeks after the treatment, and antitubercular therapy was planned to continue for one year. 

## Discussion

CNS TB, including TBM and tuberculous myelitis, poses significant challenges in diagnosis and management, often carrying an unfavorable prognosis compared to less invasive forms of tuberculosis [11]. Ensuring timely diagnosis and intervention demands an elevated level of suspicion, emphasizing the essential need for clinicians to recognize diverse clinical presentations and consider specific epidemiological contexts [3].

The conventional method for culturing mycobacteria, considered the gold standard for diagnosing TB, typically requires six to eight weeks and is consequently inadequate for the early diagnosis of TBM [13]. Moreover, the CSF in individuals with TBM frequently harbors only a limited number of organisms, leading to a generally low diagnostic yield for CSF smear and culture [13].

In the specific case outlined in this article, certain characteristics strengthen the likelihood of a tuberculous CNS infection. Factors such as a history of immunosuppression and a positive IGRA before initiating ART emerge as significant contributors. Furthermore, the CSF analysis, revealing pleocytosis with monocytic predominance, hyperproteinorrachia, and hypoglycorrhachia, strongly suggests an infection of the CNS of a tuberculous cause [4,7]. The presentation of motor deficits upon admission, along with typical signs such as fever, neck rigidity, and altered consciousness, suggested possible involvement of the spinal cord, representing a more severe form of CNS infection [11].

Notably, extremely elevated CSF protein levels suggest spinal cord involvement and correlate with poorer outcomes, emphasizing the crucial need to promptly start suitable medication [14]. Among HIV-infected patients, including those under ART, managing CNS TB presents specific considerations, including distinctions in presentation and the influence of the immunological status on the overall risk of disease and outcome [4,11,14].

Lower CD4+ cell count (<200 cells/mm³) increases the risk of TB reactivation, while higher viral loads indicate compromised immunity and a greater likelihood of severe TB manifestations, including heightened extrapulmonary and CNS involvement [14].

Some studies [9,15] have shown higher TB incidence rates in the first months of ART, decreasing significantly during the initial year of treatment, and stabilizing thereafter. Among individuals without early incident TB (defined as TB within the first three months of treatment), the risk of late incident TB was associated with treatment response, with a four-fold increased hazard for those with CD4+ cell count below 50 cells/mm³ compared to those above 350 cells/mm^3^ [9]. Furthermore, lack of viral suppression (viral load > 10,000 copies per ml) was associated with a 2.5-fold increased adjusted hazard for TB [9].

## Conclusions

TB poses a persistent threat to individuals living with HIV, even amidst ART, with the severity of HIV disease serving as a notable risk factor for both prevalent and early incident TB. Vital strides in enhancing the care of HIV-infected patients hinge on initiatives such as early ART initiation and systematic, highly sensitive screening for TB, pivotal in curbing opportunistic infection rates. The proactive diagnosis and effective management of CNS TB emerge as linchpins in diminishing morbidity and mortality while mitigating the severity of associated neurological complications.

In the reported case, the expeditious commencement of antituberculous treatment upon admission, aligned with to the patient's specific immunological status (low CD4+ cell count and moderate viral load), played a pivotal role in fostering an improved prognosis and minimizing observed neurological sequelae. The recognition of less frequently encountered signs and symptoms, especially in immunocompromised individuals like those with HIV, is imperative for early suspicion and intervention in CNS TB prognosis. Equally crucial is an understanding of CSF characteristics across various CNS infections, incorporating group-specific traits, underscoring the significance of prompt action in co-infected patients. Factors such as ART duration, CD4+ cell count, and viral load warrant careful consideration, given their substantial impact on the incidence, course, and prognosis of HIV-associated CNS TB.
